# Nickel-photoredox catalysis: merging photons with metal catalysts for organic synthesis

**DOI:** 10.1039/d5ra04650e

**Published:** 2025-09-12

**Authors:** Faiza Manzoor, Adnan Majeed, Ahmad H. Ibrahim, Muhammad Adnan Iqbal, Asma Rehman, Sadia Aziz, Anam Shahzadi, Sabahat Fatima, Sana Ejaz, Muhammad Shehroz Zafar

**Affiliations:** a Department of Chemistry, University of Agriculture Faisalabad Faisalabad 38040 Pakistan adnan.iqbal@uaf.edu.pk; b Organometallic & Coordination Chemistry Laboratory, Department of Chemistry, University of Agriculture Faisalabad Faisalabad 38040 Pakistan; c Pharmacy Department, Faculty of Pharmacy, Tishk International University Erbil Iraq

## Abstract

Nickel (Ni)-catalyzed photoredox reactions are revolutionary methods that transform organic synthesis, enabling highly efficient and selective reactions under mild conditions. The synergy between Ni catalysis and photoredox catalysis is efficacious in activating inert bonds, creating potential reaction pathways, and accessing otherwise inaccessible molecular architectures. This review provides a detailed overview of advances in nickel/photoredox dual catalysis, with particular reference to insights into mechanisms and reaction scope. Among the key developments are enantioselective allyl carbamates, *β*-phenethylamines, and aryl-*C*-nucleosides, as well as methods for hydroalkylation, aryl alkylation, and C–N/C–O coupling reactions. The single electron transfer (SET) processes and versatile oxidation states of Ni, coupled with organic and metal-based photocatalysts, underpin the dual catalytic cycles. Such innovations render Ni-catalyzed photoredox reactions more sustainable and cost-effective, providing a strong foundation for future advances in this area.

## Abstract 2

Visible-light-driven Ni-catalysis: a strategy for efficient cross-coupling.

## Graphical abstract

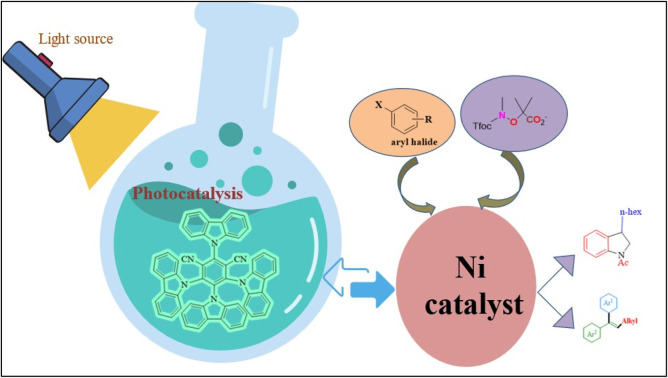

## Introduction

Catalysis has long been an essential part of modern synthetic chemistry, enabling researchers to carry out transformations that under normal conditions would be inefficient, selective, or impossible.^1^ The invention of photoredox catalysis and transition metal catalysis, two powerful methods that have recently been combined to open up new chemical innovation routes, has been one of the most important advancements in this subject.^2^ Two-electron processes like oxidative addition and reductive elimination can be reliably carried out on transition metals, especially those that can engage in numerous oxidation states. By using visible light to initiate single-electron transfer (SET) events, photoredox catalysis has made it possible to activate stable and inert compounds in mild environments.^3^ The combination of these domains, most notably the utilization of nickel with a light-absorbing photocatalyst, has resulted in a very flexible dual catalytic system that has greatly broadened the range of organic synthesis.^4^

In this dual catalysis platform, nickel in particular has become a key component because of its distinct electrical characteristics, affordable price, and wide range of reactivity. Nickel can engage in both two-electron and one-electron processes,^5^ which is advantageous compared to larger transition metals like palladium (Pd) or platinum (Pt), which frequently only operate through two-electron redox cycles.^6^ For reactions involving radical intermediates, it is the perfect partner because it can easily access a variety of oxidation states, including Ni^0^, Ni^I^, Ni^II^, Ni^III^, and rarely Ni^IV^.^7^ When combined with photocatalysts that aid in SET processes, nickel's versatility enables it to function as a dynamic and flexible center in intricate catalytic cycles.^8^ Significantly, nickel is particularly compatible with light-driven radical chemistry due to its reactivity with sp^2^ and sp^3^ hybridized substrates, tolerance for functional groups, and capacity to stabilize open-shell intermediates. These characteristics have made nickel a key component in the advancement of photoredox-based reactions, as has its abundance and smaller environmental impact when compared to noble metals.^9^

The principle of photoexcitation underlies photoredox catalysis: a photocatalyst absorbs a visible light photon and is subsequently elevated to an excited state with an increased redox potential.^10–15^ In this excited state, a single-electron transfer (SET) event can be started by the photocatalyst (PC*), giving or receiving an electron from a substrate or reagent.^16,17^ The oxidative or reductive quenching cycle may be used by the photocatalyst, depending on its design. An oxidative quenching cycle occurs when an excited photocatalyst delivers an electron to a substrate or oxidant while also becoming oxidized.^16^ It becomes reduced when it takes an electron from a sacrificial donor during a reductive quenching cycle. The neutral radicals or radical ions that are produced by SET are sometimes extremely reactive and can interact with transition metal complexes or take part in bond-forming processes.^18^ The process is catalytic in light and electron flow since the photocatalyst is renewed in the cycle by complementary redox processes.^19^

**Fig. 1:**
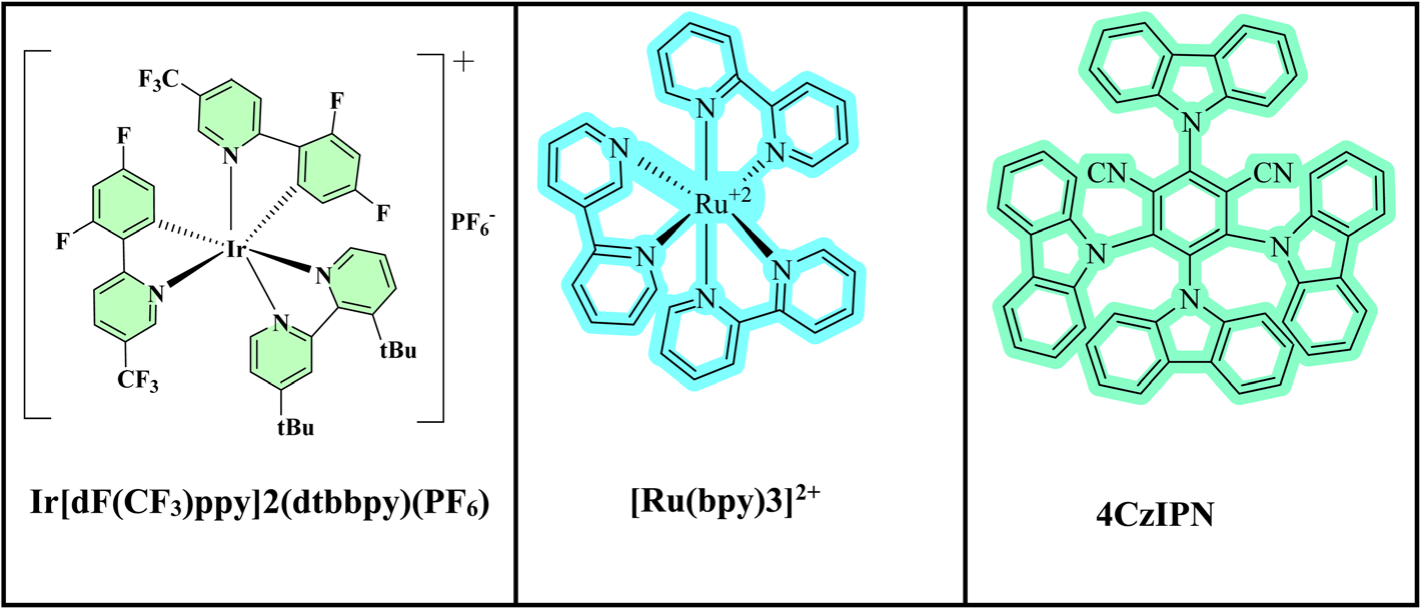
Some photocatalysts used in dual catalysis.

Metal-based and organic photocatalysts are two major categories of photocatalysts that have shown exceptional efficacy in nickel-photoredox catalysis. Transition metal complexes, most frequently based on ruthenium(ii) or iridium(iii) polypyridyl complexes (Fig. 1), are a prevalent property of metal-based photocatalysts.^20^ These substances are valued for their exceptional photostability, consistent redox activity, and extended excited states. They are versatile to a variety of substrates and reaction circumstances due to their tunable ligand environments, which enable precise manipulation of the absorption wavelength and redox potential.^21^ On the other hand, metal-free substitutes that are frequently more economical and environmentally friendly are provided by organic photocatalysts,^22,23^ such as 4CzIPN (tetra-carbazole-substituted isophthalonitrile) (Fig. 1). These organic colors are becoming more and more popular in reactions where metal contamination must be prevented,^24^ including in pharmaceutical synthesis, and can be just as effective in stimulating SET activities. The choice between the two classes of photocatalysts is usually influenced by the particular redox requirements of the reaction, substrate compatibility, and sustainability considerations. Both types of photocatalysts offer complementary advantages.^25^

A photocatalyst and a nickel catalyst work in concert to sustain both catalytic cycles through redox cycling and radical intermediates in nickel-photoredox dual catalysis. When light is absorbed, the photocatalyst (PC*) and an appropriate precursor (such as a halide, carboxylate, or organosilicate) conduct single-electron transfer (SET), producing a radical (R˙) that is caught by a nickel species (Ni^0^ or Ni^I^) to create an organonickel intermediate.^26^ An alternative approach is to use an electrophile to oxidatively add the nickel catalyst first, followed by capturing the resulting radical. In both cases, Ni^II^ or Ni^III^ initiates bond-forming reductive elimination (C–C, C–N, C–O, *etc.*), and the active nickel state is restored in a redox-neutral cycle by electron transfer from the oxidized or reduced photocatalyst. The selective creation of radicals without harsh chemicals, the softer, room-temperature conditions made possible by light energy, and the greater mechanistic adaptability, including radical capture and oxidative addition, are some of the major benefits that this dual system offers over single-catalyst techniques.^27^ By supporting both polar and radical routes, this combination increases the number of synthesis alternatives. These days, it is frequently used in both academic and industrial contexts to facilitate transformations such as complex molecule assembly, late-stage modification of bioactive chemicals, and inert bond functionalization.^28^ Air-stable nickel complexes, strong organic dyes, and electrochemical regeneration are examples of catalyst design advancements that are pushing the sector farther toward higher sustainability, scalability, and practicality.^29^

In this paper, we provide a thorough analysis of the fundamentals of nickel-photoredox catalysis, their workings, and the most recent advancements. Both metal-based and organic photocatalysts are highlighted, along with the general mechanisms of dual catalysis and the special contributions of nickel as a key element in these systems. In order to drive chemical reactivity in novel and potent ways, we seek to present a comprehensive and integrative view of the interactions between light, electrons, and transition metal complexes. This review emphasizes the broad applicability of nickel-photoredox dual catalysis, the variety of catalytic strategies employed, and the fundamental concepts that continue to drive innovation in this rapidly developing area of synthetic chemistry. Rather than concentrating only on particular bond types or substrate classes, we examine the philosophical underpinnings, underlying mechanisms, and synthetic possibilities of nickel-photoredox catalysis, with special attention to its compatibility with both metal-based and organic photocatalysts.

While several earlier reviews (2014–2021) have summarized the foundations of nickel-photoredox catalysis,^30–33^ our work distinguishes itself by emphasizing breakthroughs reported between 2022 and 2025. In particular, we highlight emerging mechanistic paradigms such as proton-coupled electron transfer (PCET)^34^ and radical-polar crossover processes, as well as underexplored substrate classes including strained heterocycles,^35^ ribosyl acids, and oxetanyl building blocks.^36^ This updated perspective not only surveys the newest synthetic methodologies but also integrates recent mechanistic advances that were not covered comprehensively in previous reviews.

## Ni/Organophotoredox catalysis

**Scheme 1:**
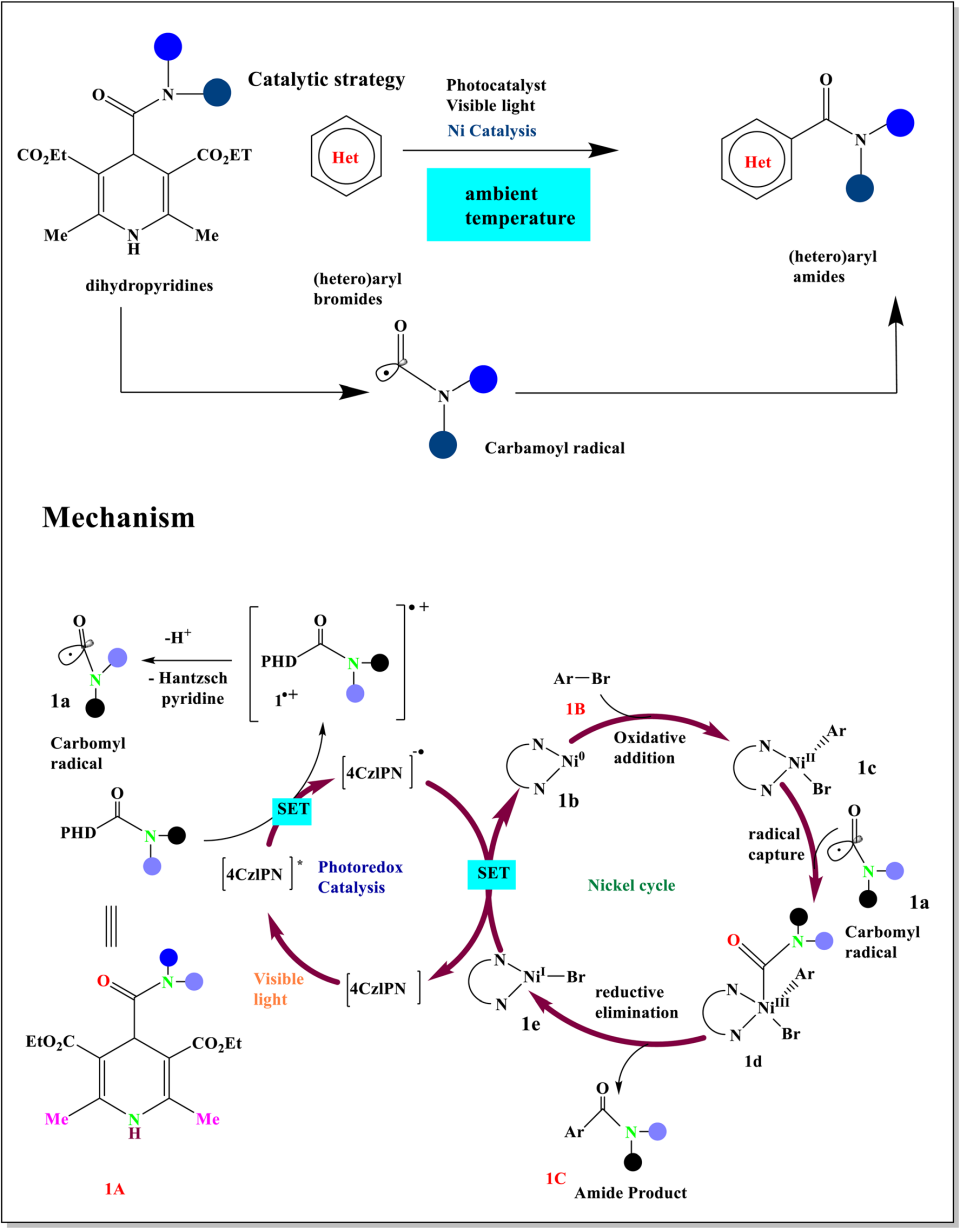
Proposed mechanism for the carbamoylation process catalyzed by photoredox and Ni catalysts.

Nurtalya and coworkers described a one-electron method for catalytic amide (1C) synthesis that uses photoredox and nickel dual catalysis^5^ to allow for the direct carbamoylation of (hetero) aryl bromides (1B).^4^ The ability of the nickel catalyst to participate in radical capture activities and undergo oxidative addition with aromatic bromides 1B guaranteed the synthesis of the cross-coupled amide products 1C. The organic photocatalyst 4CzIPN is photoexcited,^37^ creating an oxidant potent sufficient to absorb an electron from the precursor of the radical 1A.^38^ The production of the carbamoyl radical 1a is triggered by this SET event (Scheme 1). The Ni^0^ complex 1b is simultaneously oxidatively added to aromatic bromide 1B to form the Ni^II^ complex 1c. Intercepting the nucleophilic carbamoyl radical 1a with the Ni^III^ intermediate 1d results in the creation of the cross-coupled amide product 1C. It subsequently produces the necessary C(sp^2^)–C(sp^2^) bond through reductive elimination. After that, the reduced form of the photoredox catalyst performs SET^8^ reduction on the resultant Ni^I^1e intermediate to finish the catalytic cycle and replenish both active catalysts.^39^

**Scheme 2:**
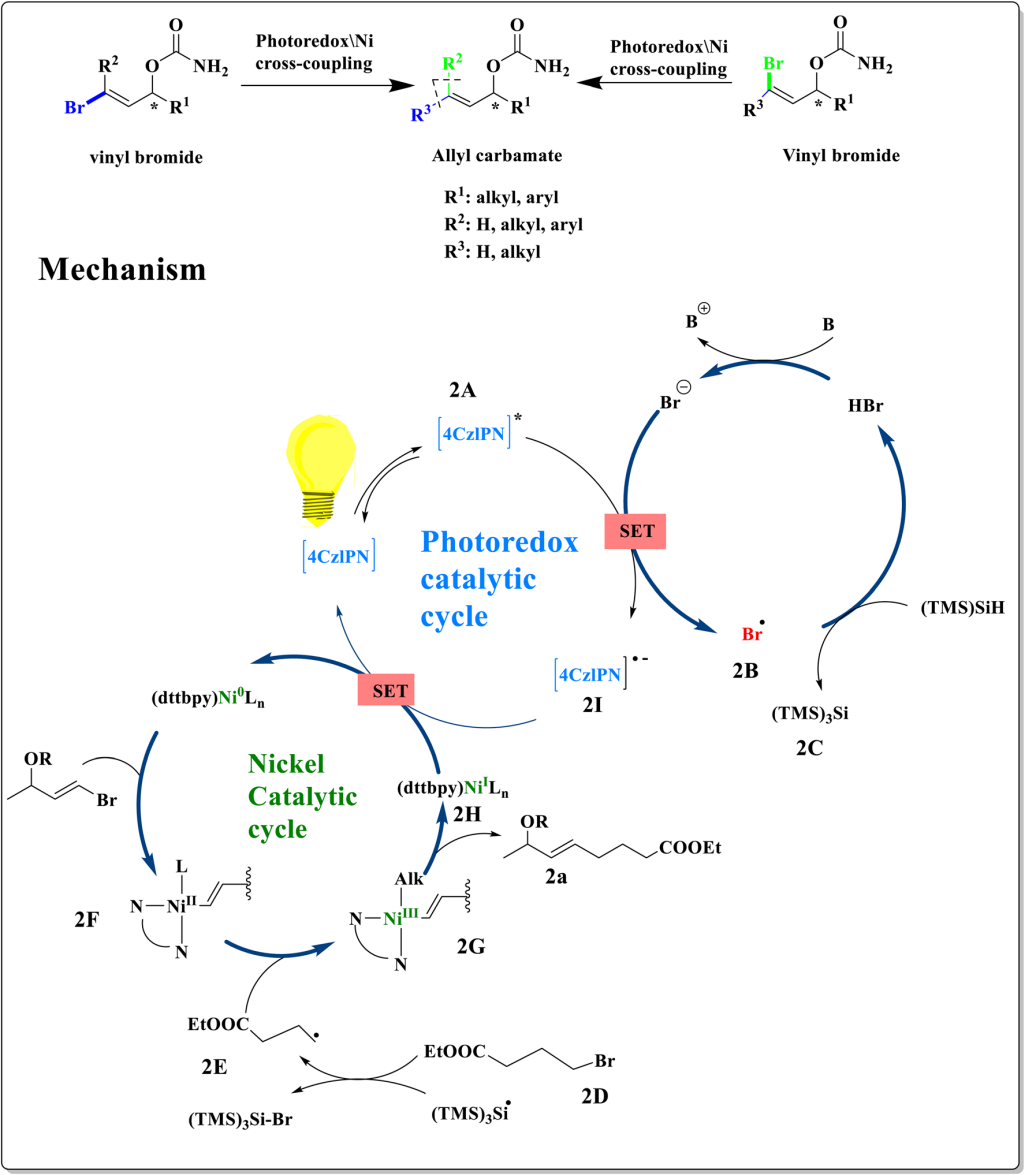
Proposed mechanism for photocatalytic alkenylation of vinyl bromides. ^*a*^ L= ligand.

Enantioenriched allyl carbamates were developed by using a dual photoredox/Ni-based strategy. 4CzIPN absorbs photons^40^ to excitations to the powerfully oxidizing agent [4CzIPN]* (2A) when exposed to LED light.^41^ To remove a hydrogen atom from^18^ (TMS)_3_SiH, this complex can oxidize the bromide anion (2B) to form a bromine radical.^10^ Ni^0^ complex can be added oxidatively to vinyl bromide on its own to produce an intermediate (2F). The alkyl-Ni^III^ complex (2G) is then produced by simple oxidative radical capture. Ni^I^ species and the C(sp^3^)–C(sp^2^) coupling product,^2,42^ such as 2a, obtained by reductive elimination from (2H). Finally, the latter reduced to Ni^0^, and a single electron transfer from the accessible species to the Ni^I^ complex (2I) restored the ground state of the photocatalyst^21^ (Scheme 2).

**Scheme 3:**
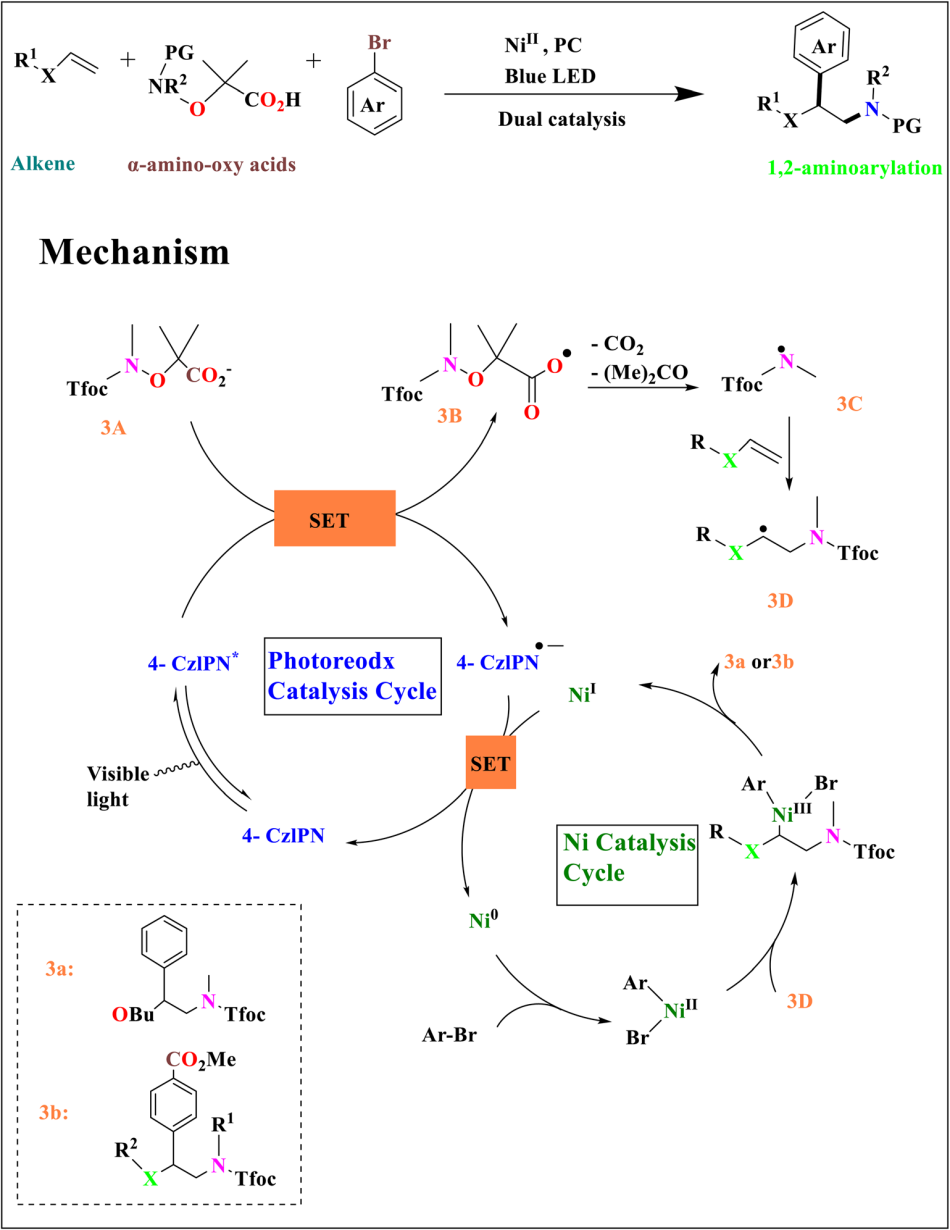
Proposed catalytic cycle for the synthesis of *β*-phenethylamines.

By using nickel catalysis and synergistic photoredox, Heng Jian and coworkers described a three-component 1,2-aminoarylation of vinyl ethers, enamides, ene-carbamates, and vinyl thioethers. The photoexcitation of 4-CzIPN by radiation is the initial step in the catalysis cycle. This causes an excited redox catalyst to oxidize carboxylate 3A,^6^ forming reduced 4-CzIPN and the carboxyl radical 3B.^41^ An electrophilic N-radical 3C is created by the successive fragmentation of acetone and CO_2_.^41^ This radical then reacts with alkene to form 3D. Ni^I^ completes the photoredox cycle by oxidizing CzIPN, resulting in a Ni^0^ species that oxidatively joins the aryl bromide to form an intermediate Ni^II^–Ar. The Ni^III^ species 3E is created when the Ni^II^–Ar complex captures the radical 3D. The nickel catalysis cycle is closed when 3a or 3b is linked with a Ni^I^ molecule through reductive elimination.^39^ Aminoacylation functions similarly by substituting the acylated succinimide for the bromoarene^34^ (Scheme 3).

**Scheme 4:**
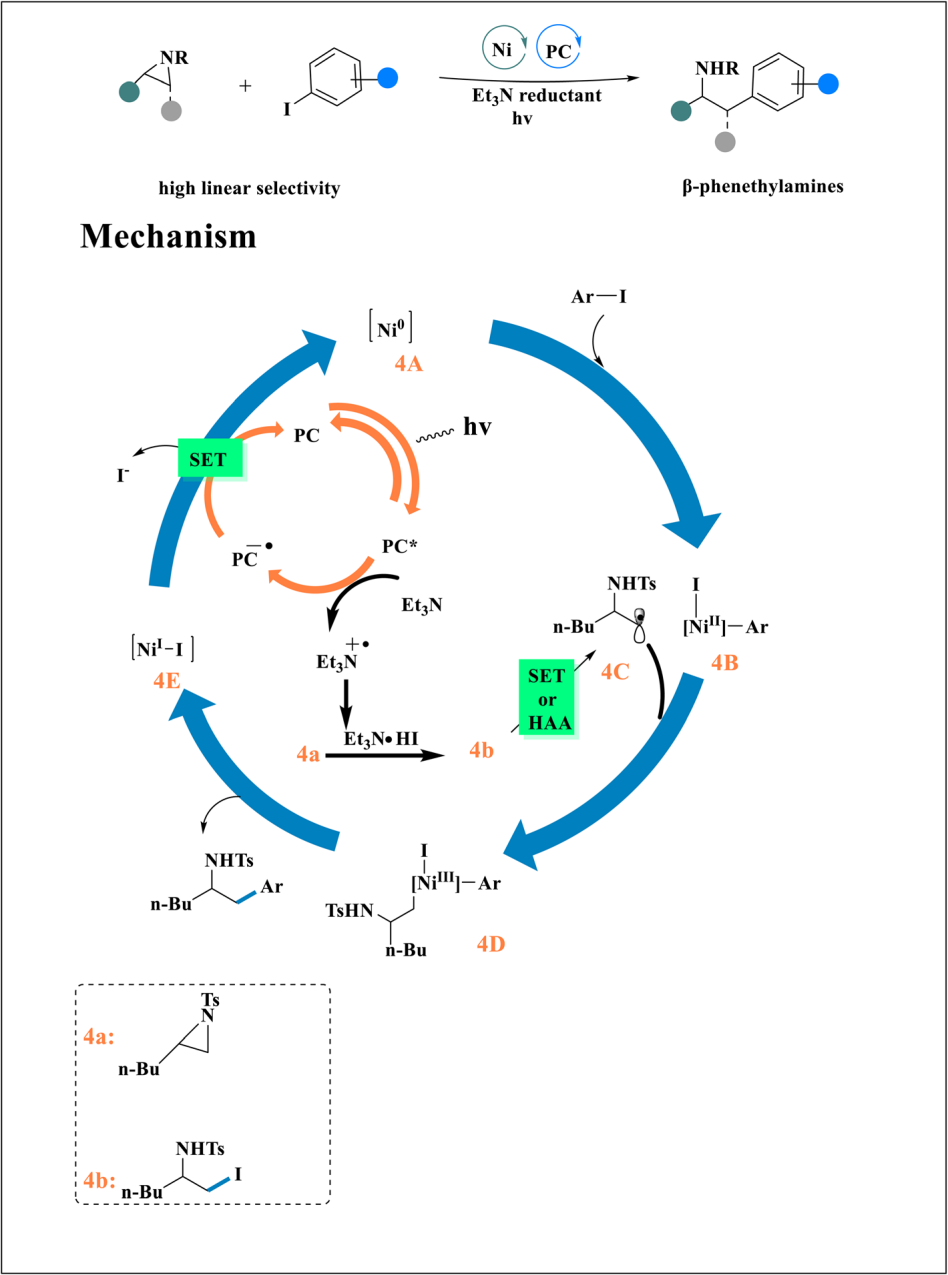
Proposed mechanism for the synthesis of *β*-β-phenethylamines.

Tosyl-protected alkyl aziridines and (hetero)aryl iodides underwent a photoassisted Ni-catalyzed^24^ reductive cross-coupling to synthesize *β*-phenethylamines. The 4B was created by the facetious oxidative addition of aryl iodide to Ni^0^. Concurrently, the nucleophilic iodide ring opening of 4a to become 4b was mediated by the *in situ* synthesis of HI. The SET of 4b (using [dtbbpy]Ni^I^–IE or 4CzIPN^−^˙), or halogen atoms abstraction (HAA) from 4E produced 4C.^24^ With 4B, this radical intermediate can be trapped. Reductive elimination from the synthesis of 4D yields the cross-coupled product and intermediate 4E, which is then reduced to 4D by the 4CzIPN^−^˙ (Scheme 4).^43^ A process involving the selective addition of Ni^I^–IE to the aryl iodide, single-electron reduction, and reactivity of the resulting Ni-aryl intermediate with iodoamine^27–29^4b is proposed^37^ in addition to the Ni^0^/Ni^II^/Ni^III^/Ni^I^ cycle.

**Scheme 5:**
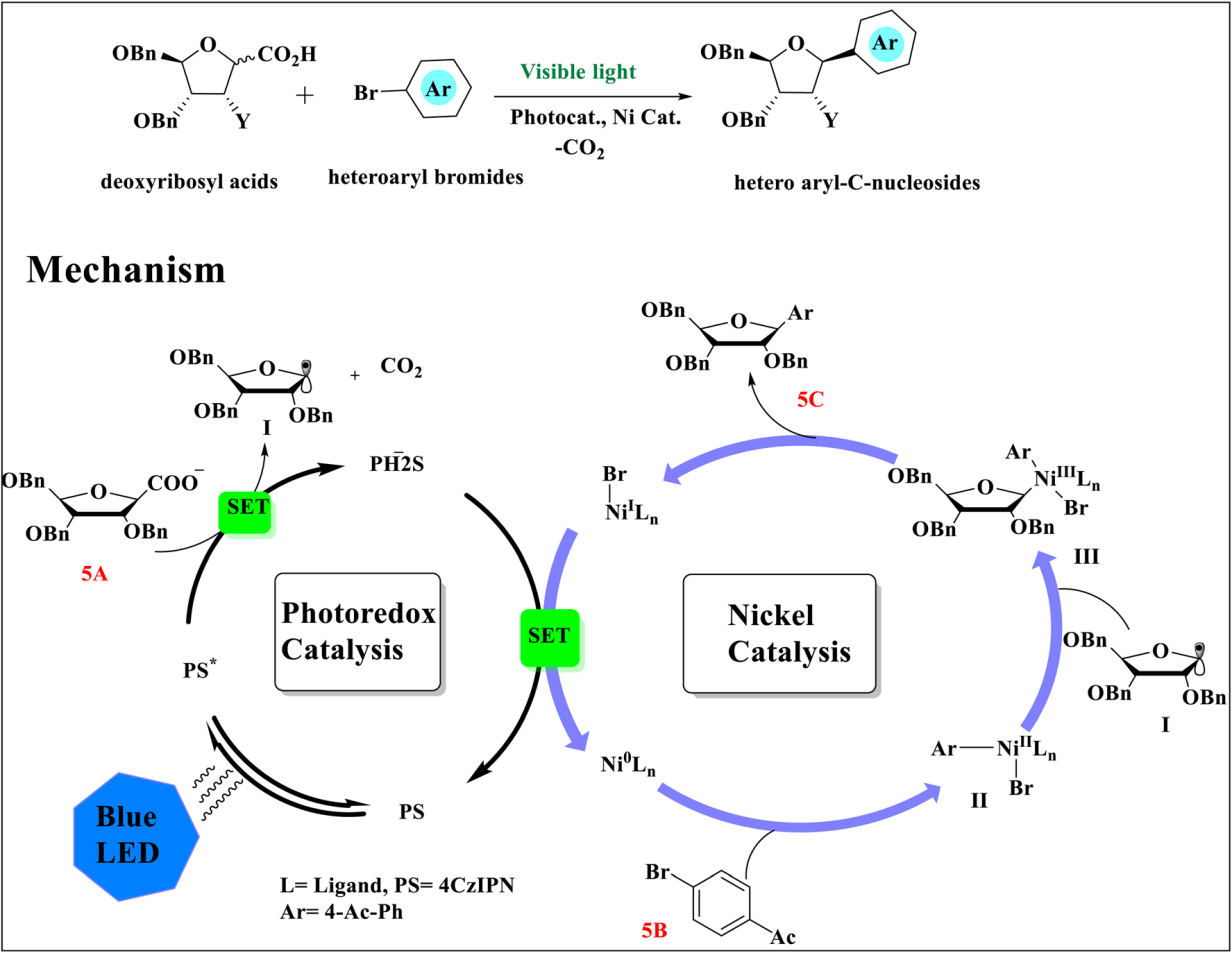
Proposed mechanism for the preparation of nucleosides.

Instead of photoredox-catalyzed decarboxylative cross-coupling, anomeric ribosyl/deoxyribosyl acids and aryl/heteroaryl bromides were converted to aryl\hetero-aryl-*C*-nucleoside. Initial excitation results in the production of the 4CzIPN (PS) in a photoexcited state (PS*).^41^ The production of a sp^2^ hybridized anomeric radical I and the photooxidative decarboxylation processes of 5A is made possible by the high reduction potential of the photoexcited state^37^ of 4CzIPN (PS*).^44^ The electrophilic Ni^II^-aryl intermediate II is expected to be created in combination with that photoredox cycle by oxidatively adding to the aryl bromides 5B by two SET reductions of (bpy)Ni(ii)Br_2_ produced by the photocatalyst PS, generating active Ni^0^ species (Ni^0^Ln) *in situ* (Scheme 5). This Ni^II^ species produced a Ni^III^-aryl-ribosyl complex III by quickly capturing anomeric ribosyl radical I. Following reductive elimination, this combination produced the Ni^I^ complex (bpy)Ni^I^Br and the intended product 5C.^34^

Achieving rapid and extremely selective amidoarylation of inactivated olefins using photoredox proton-coupled transfer of electrons revealed the crucial equilibrium between kinetically-driven cyclization and thermally-driven radical production. Amidyl radical 6B is formed by PCET to start the mechanism, as demonstrated by Stern–Volmer analyses, NMR testing, and cyclic voltammetry.

**Scheme 6:**
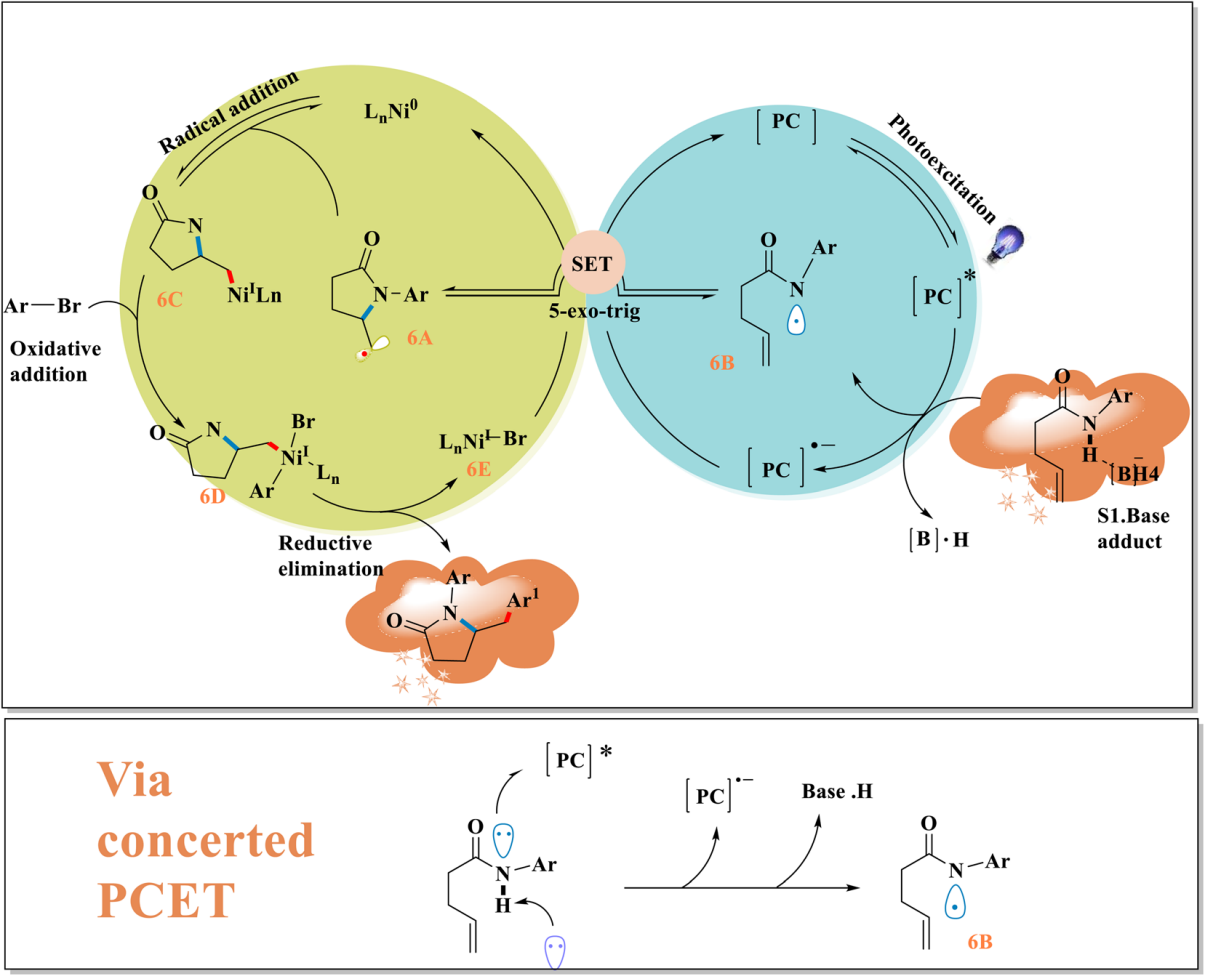
Proposed catalytic cycle with PCET mechanism.

This was followed by a quick 5-*exo*-trig cyclization.^41^ This is correlated with the type of freshly generated alkyl radical and N-HBDE, by indirect kinetic investigations (Scheme 6). The aryl halide undergoes oxidative addition by the Ni^I^-complex 6C, which is formed when the alkyl radical 6A enters the nickel-catalytic cycle. Following reductive elimination of the resulting high-valent Ni^III^ intermediate 6D, the end product 1 and a Ni^I^-halide complex 6E are obtained. Both catalytic cycles are simultaneously closed when the reduction of Ni^I^ halide takes place using the reduced state of the photocatalyst.^43^

Huaigui Li created a photoredox/Ti dual-catalyzed dehydroxylative ring-opening Giese reaction of cyclobutanone oximes in order to avoid oxime prefunctionalization and stoichiometric phosphines that demonstrated a broad range and moderate conditions.^45^ Mechanistically, Cp_2_TiCl_2_ quenches photoexcited 4CzIPN to produce Cp_2_Ti^III^Cl, which causes N–O cleavage to produce iminyl and *γ*-cyanoalkyl radicals. When they are added to *N*-acrylamides, the product is obtained by HAT from Hantzsch ester, and the Ti cycle is concluded through the regeneration of Cp_2_TiCl_2_.^46^ Longzhou Qin and coworkers used 4CzIPN, DBU, and DMSO to create a metal-free, environmentally friendly photoredox decarboxylative alkynylation process for carboxylic acids with alkynyl bromides.^47^ It is adaptable to batch and flow systems, produces more than 50 samples with quick reaction times, and operates in moderate, eco-friendly conditions.^48^ Mechanistically, bromine radicals are reduced to replenish the catalyst in the process of photoredox decarboxylation of carboxylates to alkyl radicals, which then combine with alkynyl bromides to create the product.^49^

## Ni/Metallaphotoredox catalysis

**Scheme 7:**
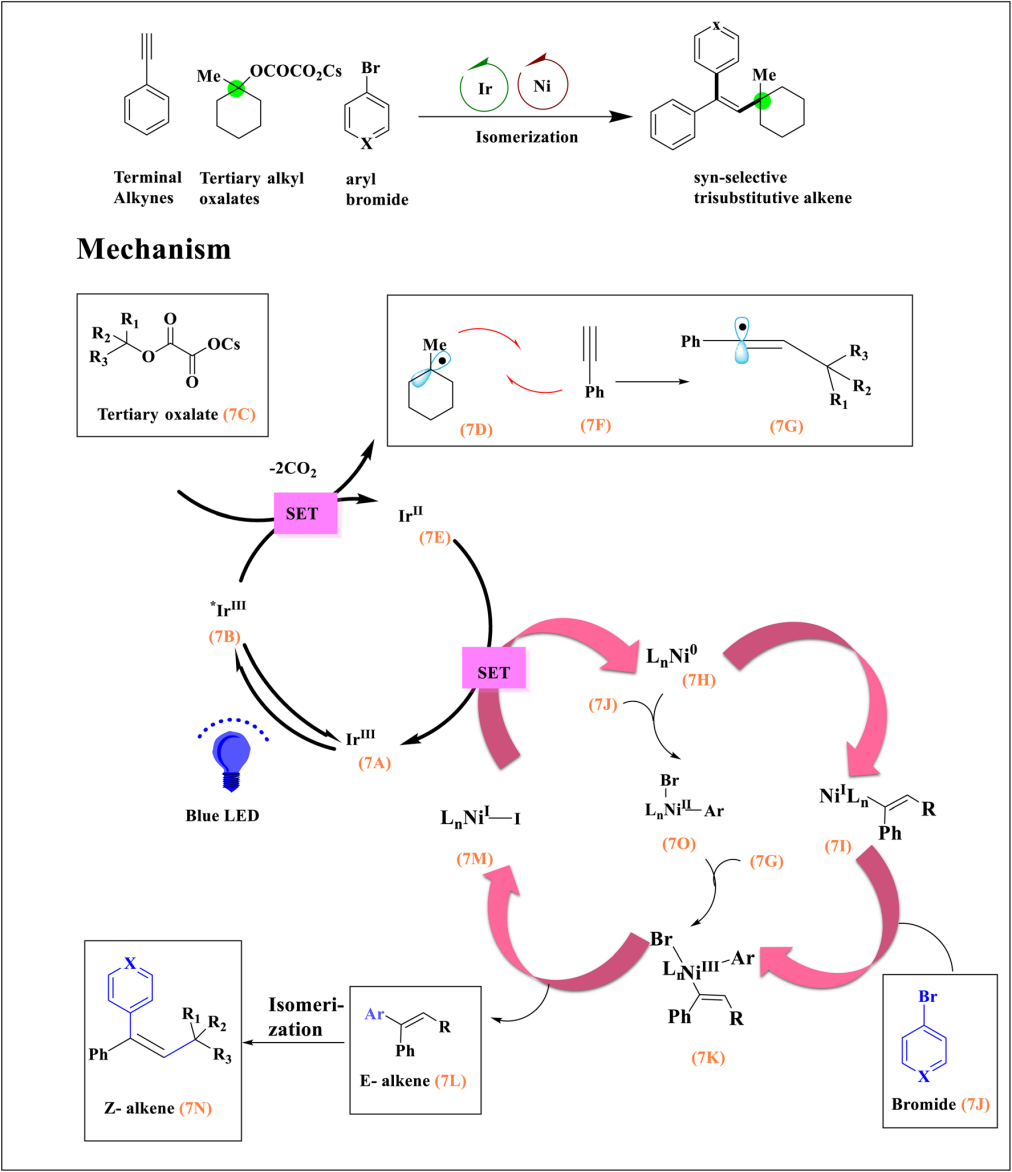
A suggested method for using photoredox and nickel catalysts to *syn*-selectively alkylate terminal alkynes.

Highly efficient and *syn*-stereo-selective trisubstituted alkenes were generated by combining photoinduced alkene isomerization with Ir/Ni-catalyzed alkyne difunctionalization. Photoexcited Ir[dF(CF_3_)ppy]_2_(dtbbpy)(PF_6_) (7A) and a single-electron oxidation of tertiary alkyl oxalate (7D) (ref. 50) by reducing Ir^II^7E species^51^ and losing two molecules of CO_2_ (Scheme 7). Regioselective addition of alkyl radical (7D) to terminal alkyne (7F) resulted in linearized alkenyl radical (7G) due to the stabilizing effect.^51^ (*E*)-Alkenyl-Ni^I^ species (7I) were created by antiaddition between this high-energy radical 7G and Ni^0^7H.^52^ Oxidative addition of 7I and 7J affords (*E*)-alkenyl-Ni^III^ complex (7K),^50^ which, upon facile reductive elimination, delivers substituted alkene (7L) and Ni^I^ complex (7M). Two catalytic cycles were closed by single electron transfer between Ir^II^7E and Ni^I^7M by reproducing ground state 7A and Ni^0^. Photoinduced energy transfer causes *E* → *Z* isomerization of 7L.^53,54^ The key for Ni^III^ intermediate 7K can also be obtained by an alternate catalytic pathway that entails the oxidative addition^51,52^ of Ni^0^ with arylbromide 7J and the trapping of the nucleophilic vinyl radical 7E by aryl-Ni^II^ (7O).^55^

**Scheme 8:**
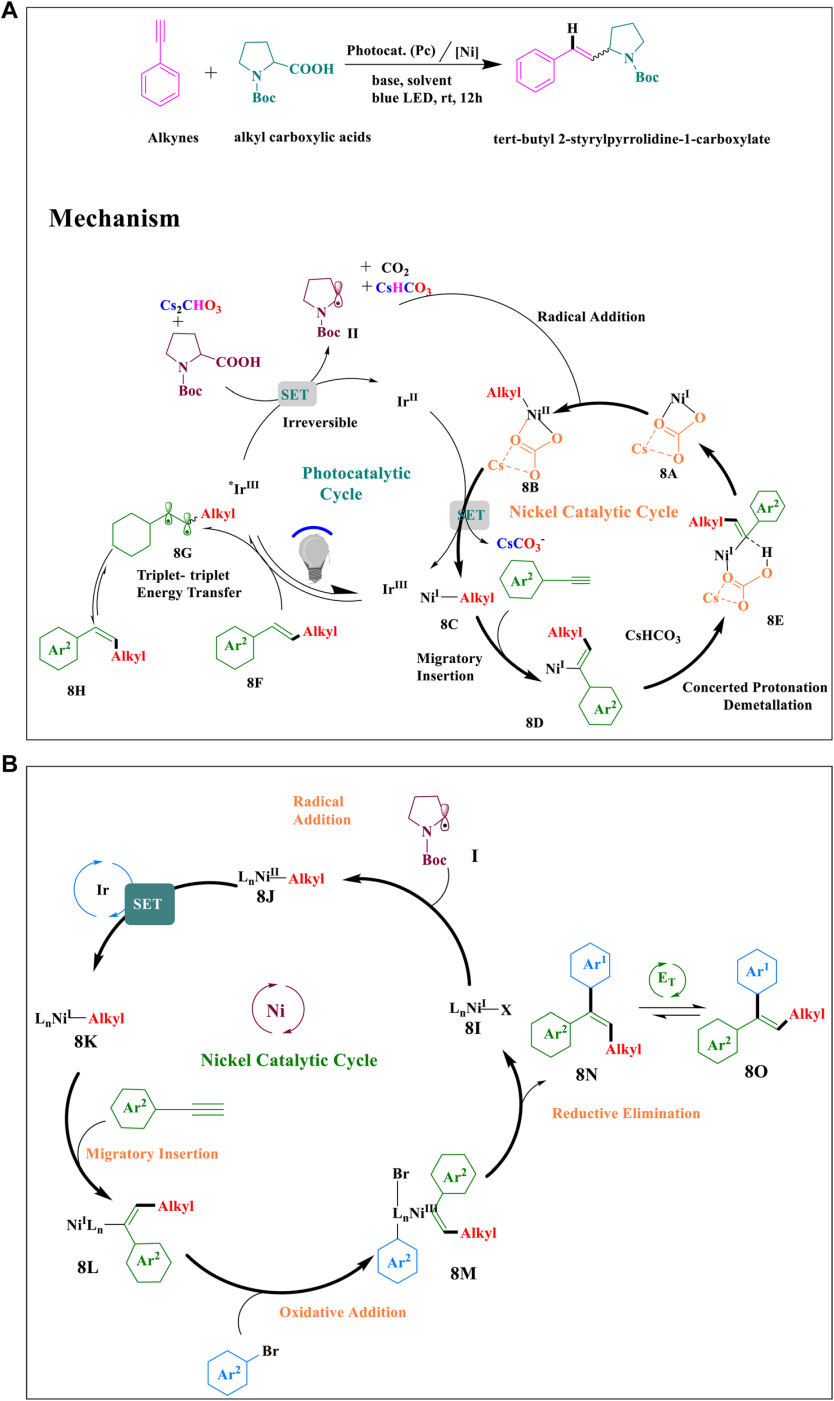
(a) Proposed mechanism for the hydroalkylation of alkynes by photoredox and nickel dual catalysis. (b) A proposed mechanism for the arylalkylation of alkynes by photo/nickel dual catalysis.

A one-pot arylalkylation of alkynes with alkyl carboxylic acids and aryl bromides by a three-component cross-coupling, as well as anti-Markovnikov-type hydroalkylation of terminal alkynes, were reported by Huifeng Yue and colleagues using photoredox/nickel dual catalysis. First, visible light is absorbed by the Ir^3+^ photocatalyst (Scheme 8a), creating a triplet excited state (*Ir^3+^) that lasts for a long time. After the carboxylic acid is oxidized by the excited *Ir^3+^ species, CO_2_ is extruded and an intermediate alkyl radical II is formed^42^ and Ni^I^ complex (8A) traps it to generate Ni^II^ intermediate (8B).^56^ Ni^I^ intermediate (8C) is produced as a result of the reduction of 8B by Ir^II^ reductant, which, upon migratory insertion, develops Ni^I^ intermediate (8D).^55^ Through a Concerted Protonation–Demetallation (CPD) process (8D), is protonated *via* intermediate (8E) in the hydroalkylation route, producing the *E*-isomer (8F) and regenerating the Ni^1+^ complex.^57^ Through an energy transfer pathway, the *Z* isomer is obtained from the *E* isomer (8F) *via* intermediate (8G).^58^

In case of arylalkylation, the aryl halide undergoes oxidative addition (Scheme 8b) in the generated Ni^I^ intermediate (8L) to produce intermediate (8M), which is removed reductively to generate the anti-addition three-component coupling product (8N).^58^ The final result is the *syn*-addition three-component binding product (8O), which is created *via* an energy transfer mechanism.^58^

**Scheme 9:**
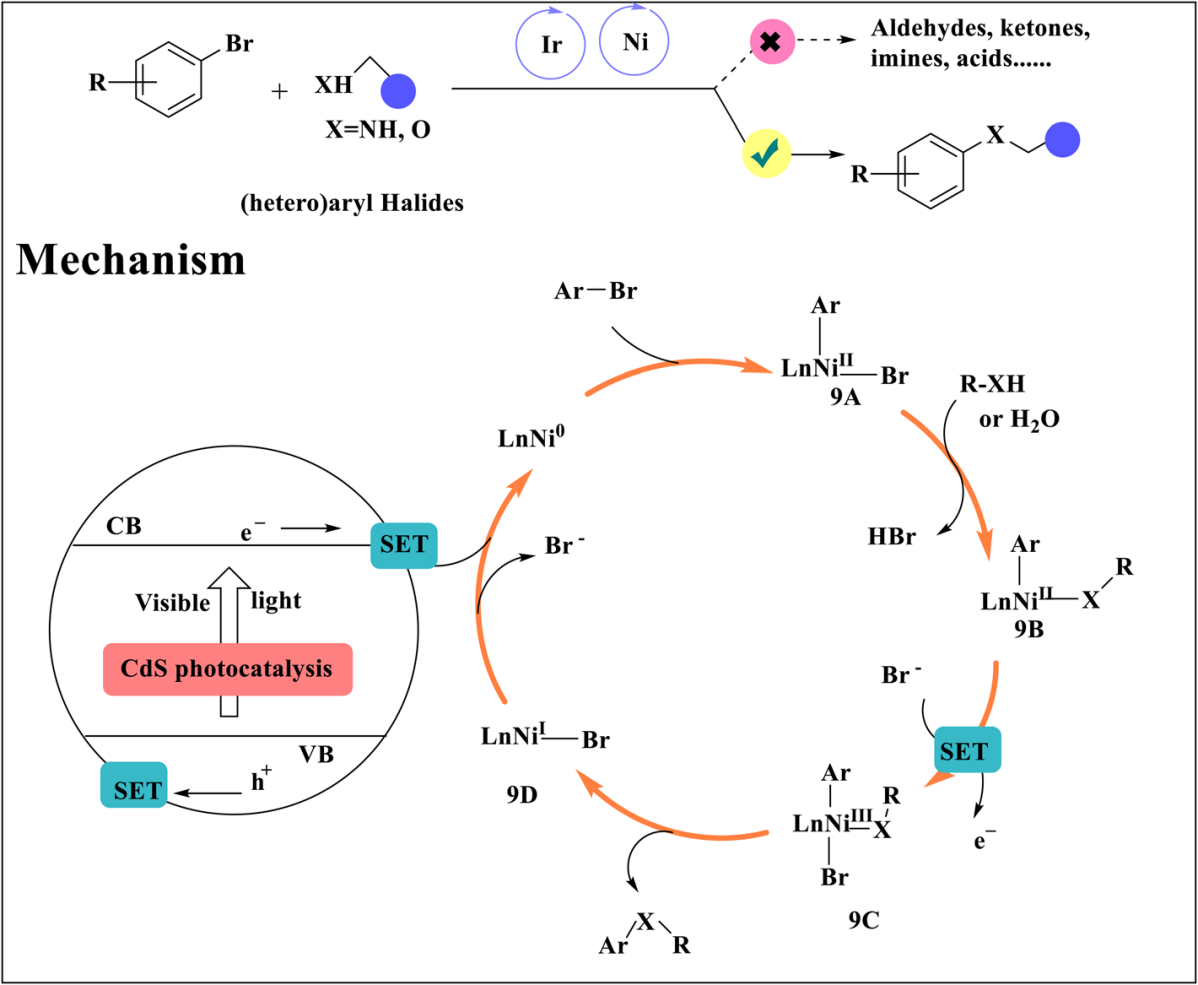
Proposed mechanism photo/nickel dual-catalyzed C–N/C–O coupling.

Effective C–N and C–O coupling reactions of aryl halides with amines and alcohols have been developed using the nickel dual catalysis method and heterogeneous visible light photoredox, which has made them appealing to the synthetic community.^51^ Aryl bromides are oxidatively added by a Ni^0^ catalyst, producing an aryl Ni^II^ species 9A, which is then converted into an aryl Ni^II^ intermediate 9B through ligand exchange with amines, alcohols, and water (Scheme 9). Visible light is used to excite the heterogeneous photocatalyst CdS. The oxidizing holes in the valence band (VB) of photoexcited CdS abstract an electron from the Ni^II^ species 9B to produce species 9C. This species is expected to undergo reductive elimination and produce aryl amines, ethers, and phenols. When the conduction band of photoexcited CdS provided an electron to the active Ni^0^ species, reducing the Ni^I^ to complex 9D, the catalysis cycle was completed.^51^

**Scheme 10:**
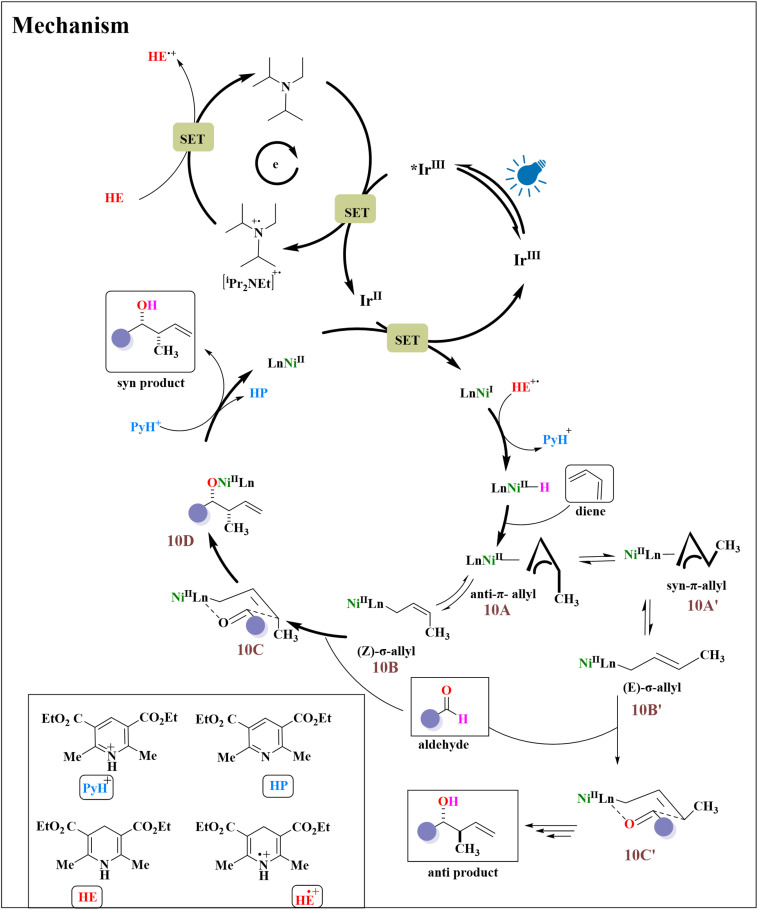
Synthesis and proposed mechanism for homoallylic alcohols.

Yan Lin and coworkers used visible light photoredox dual catalysis to describe the Ni-catalyzed reductive coupling of aldehydes and 1,3-diene. Ir^III^ photocatalyst is excited to produce the photoexcited Ir^III^ intermediate. Pr_2_NEt has reduced Ir^III^ catalyst *via* SET to generate Ir^II^ species, which has taken one electron from Hantzsch ester (HE) to regenerate Pr_2_NEt and radical (HE˙^+^).^51^ Ir produces active Ni^I^ species^59^ and Ir^III^ through the reduction of the ligand-coordinated Ni^II^ complex, which closes the iridium photocatalytic cycle (Scheme 10). After a hydrogen radical is captured from HE using species, nickel hydride (Ni–H) species and pyridium ions (PyH) are produced. Hydrometalation of nickel hydride with s-cis conformer of 1,3-diene generated intermediate 10A,^60^ rapidly isomerizes to 10A′. After that, (*Z*) and (*E*)-*o*-crytol intermediates 10B and 10B′ are created. *Syn*-product is generated by Zimmerman–Traxler transition state 10C by C–C bond formation.^59^ PyH^+^ caused protonation of 10D, has gave homoallylic alcohol product.^61^
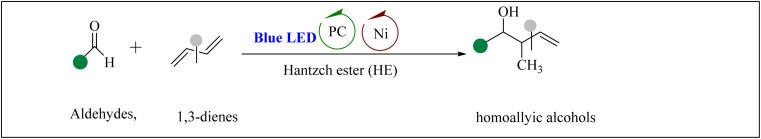


**Scheme 11:**
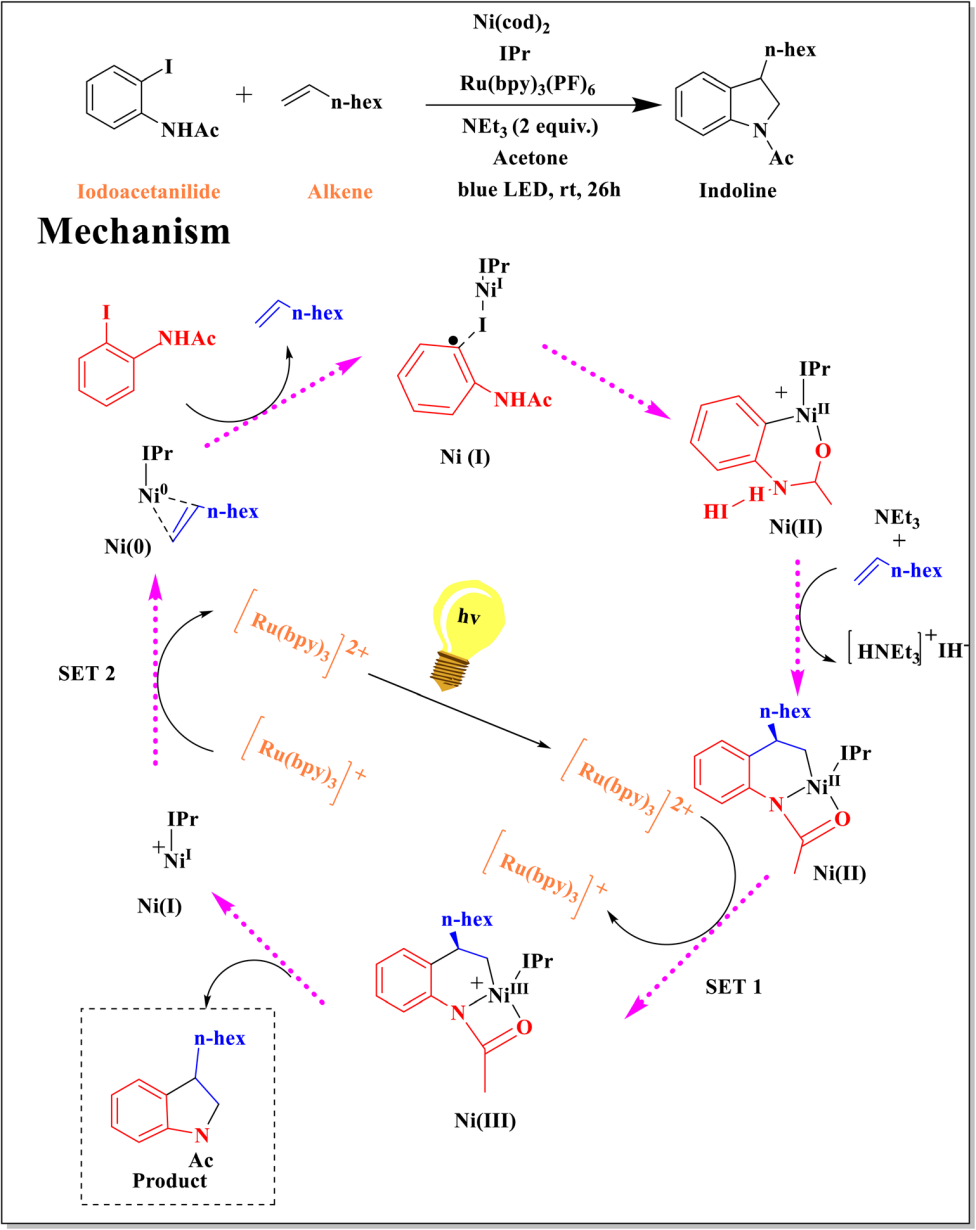
Calculated pathway for the full catalytic cycle of indoline formation catalyzed by [IPrNi] and Ru photocatalyst.

Adiran and coworkers produced indoline by combining a nickel complex with a photoactive ruthenium species to catalyze the reaction between iodoacetanilide and an alkene (Scheme 11). The dark cycle has generated a C–C bond and activated the ioacetanilide and alkene substrates by switching between Ni^0^, Ni^I^,^62^ and Ni^II^. The productive Ni^III^ species, which undergoes reductive elimination and liberates the indoline product, is created when the Ru-based photoredox catalyst oxidizes the Ni^II^ intermediate during the light cycle.^63,64^

**Scheme 12:**
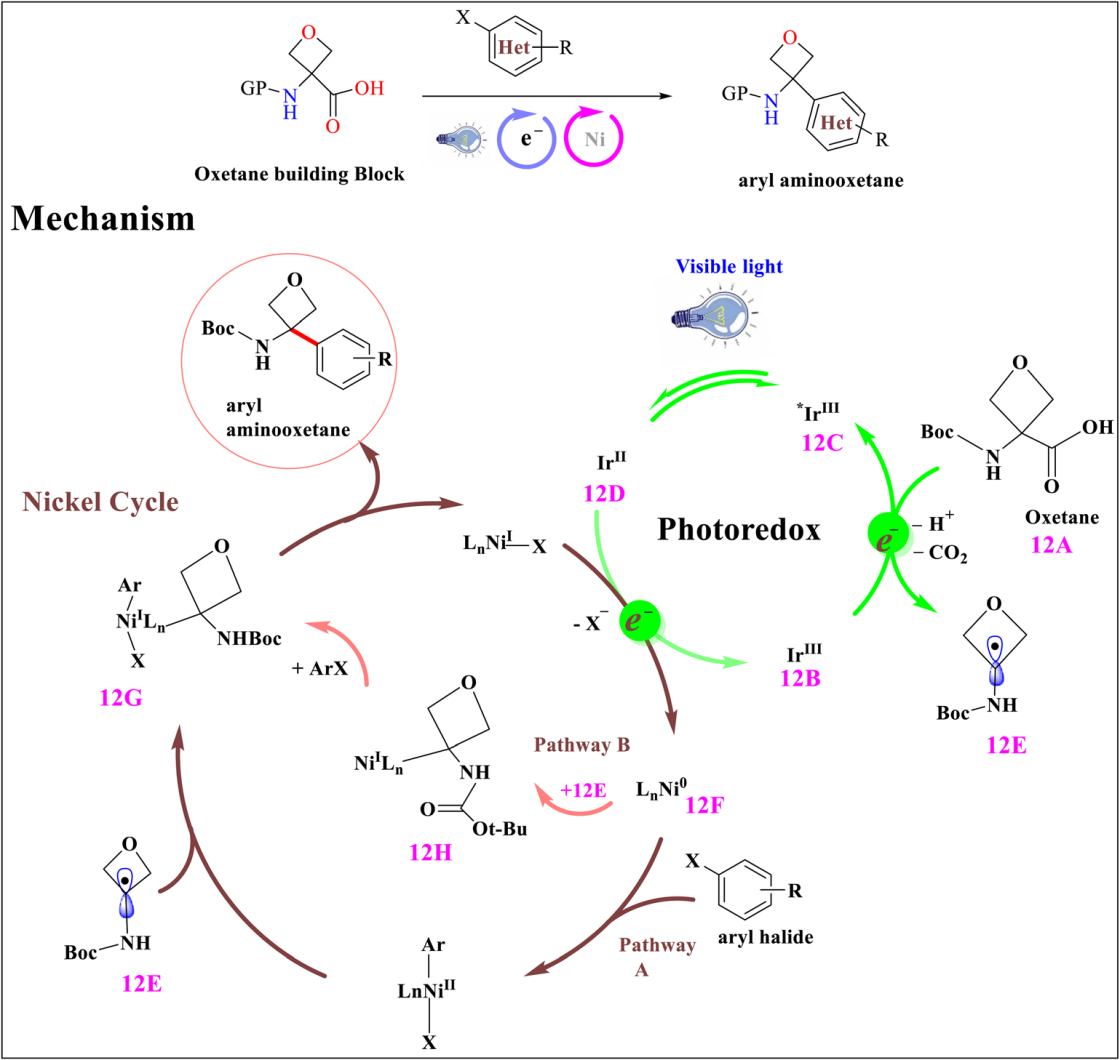
Proposed mechanism for oxetanylation of arylhalides using two distinct nickel routes.

Nickel and photoredox catalysis use light to generate chlorine radicals, which activate C(sp^3^)–H bonds for cross-coupling, without needing harsh reagents. This enables selective, mild C–C bond formation in complex molecules. Aryl halides were converted into aryl aminooxetanes by dual photoredox/Ni catalysis. Visible-light irradiation initially excited Ir^III^12B, resulting in the long-lived *Ir^III^ excited state 12C. The significant oxidizing potential of *Ir^III^ allowed the oxidation of oxetanyl amino acid 12A to the matching carboxyl radical. That delivered Ir^II^12D and key oxetanyl radical 12E, followed by quick decarboxylation. The active Ni^0^ species 12F is created by two-electron reduction of the nickel^II^ precatalyst [Ni (dtbbpy)(H_2_O)_4_]Cl_2_, initiating the nickel catalytic cycle. The mechanism proceeds forward in two different ways at this point, as described by Molander/Kozlowski and Doyle/MacMillan, respectively. Following pathway A, Ni^III^ aryl alkyl species 12G might be created by first adding Ni^0^ oxidatively to an aryl halide and combining it with oxetanyl radical 12E. Oxetanyl radical 12E was added to Ni^0^ in the B pathway to create Ni^I^ alkyl 12H, which was then subjected to ArX oxidative addition to yield the identical Ni^III^ species 12G. Both mechanisms resulted in the reductive removal of Ni^I^ and the required aryl oxetane product. SET event is used to close both of the suggested catalytic cycles, regenerating Ir^III^ and Ni^0^, respectively^36^ (Scheme 12).

**Scheme 13:**
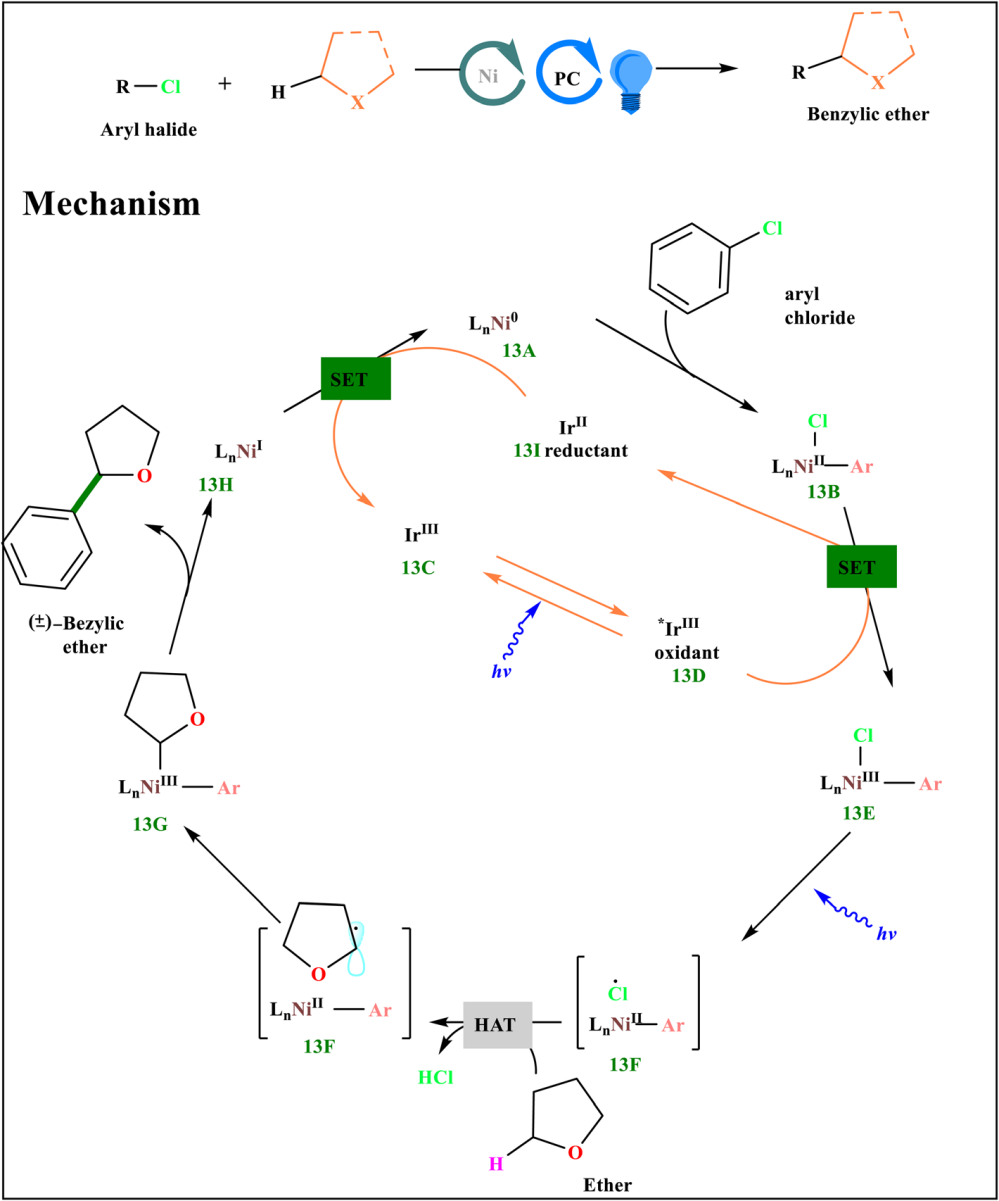
A proposed catalytic cycle for chlorine photoelimination-based arylation of ethers.

An aryl chloride is oxidatively treated with Ni^0^ complex 13A to get Ni^II^ aryl chloride intermediate 13B. At the same time, iridium^III^ photocatalyst 13C is exposed to radiation,^56,65^ which oxidizes Ni^II^ intermediate 13B to 13E and creates a long-lived, highly oxidizing *Ir^III^ triplet excited state 13D. A photon of visible light then homolyzed the Ni^III^-chlorine link, producing a chlorine radical and Ni^II^ aryl species 13F. This is because the bond is weak enough. Ni^III^ species 13G are created when the carbon-centered radical bounces back into 13F, and a hydrogen atom has been extracted from THF by the photocatalytically generated chlorine radical (Scheme 13). After reductive elimination of Ni^III^,^65^ Ni^I^ species 13H, and a new C(sp^3^)−C(sp^2^) link would arise. Then, to replenish the Ni^0^ and Ir^III^ catalysts, they would be severely reduced by Ir^II^ species 13I.^52^

**Scheme 14:**
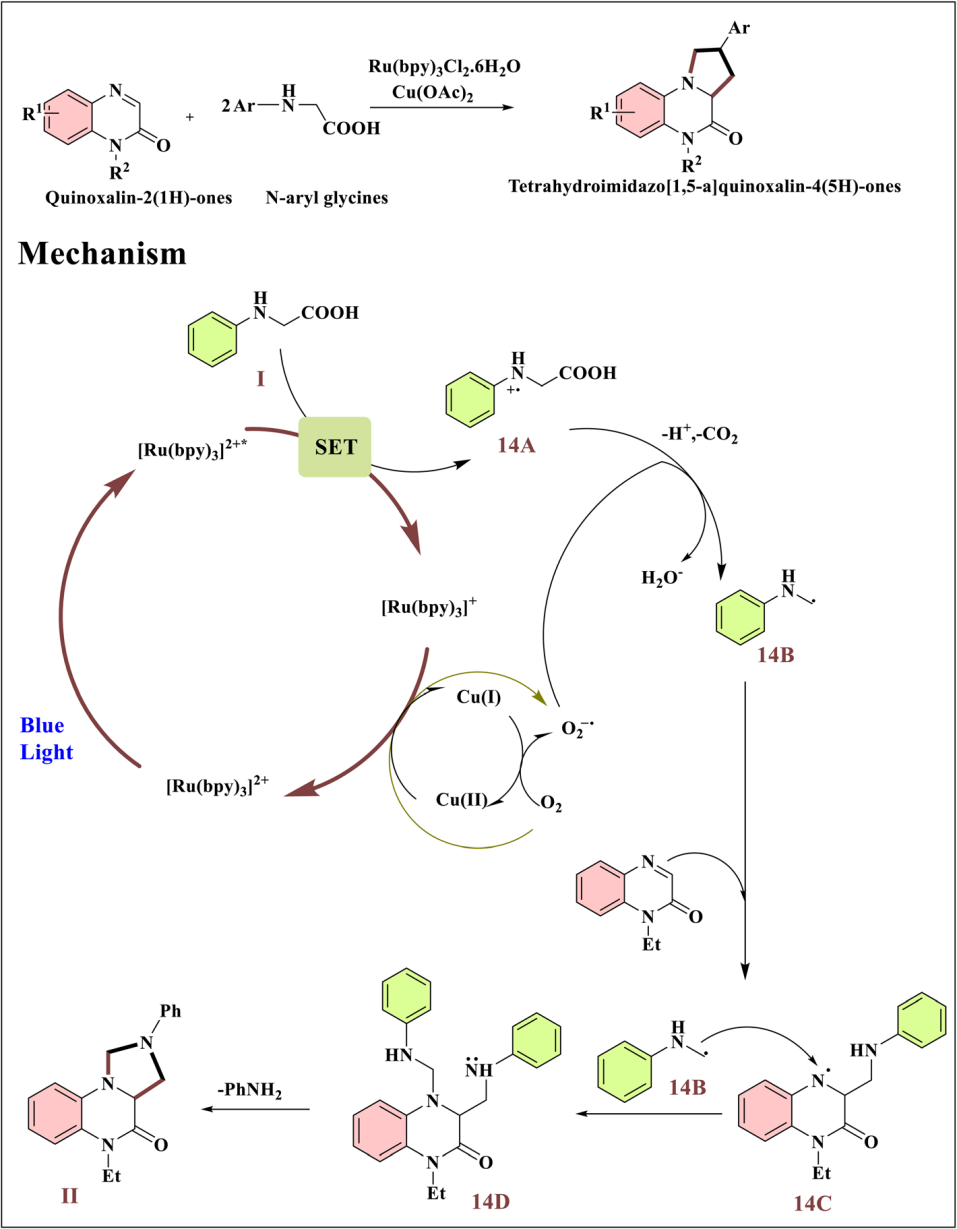
Ru/Cu dual photoredox catalysis drives decarboxylative cyclization.

Zhen Tang and colleagues reported a formal [2 + 2 + 1] cyclization of *N*-aryl glycines with quinoxalin-2(1*H*)-ones driven by visible light, which yielded tetrahydroimidazo[1,5-*a*]quinoxalin-4(5*H*)-ones.^66^ Using blue LED light, O_2_/Cu(OAc)_2_ as oxidants, and Ru(bpy)_3_Cl_2_·6H_2_O as a photocatalyst, the approach provides moderate conditions with a wide range of substrates. Glycine I is oxidized to radical cation 14A by the excited [Ru(bpy)_3_]^2+^*, producing [Ru(bpy)_3_]^+^, which is then reoxidized by O_2_ or Cu(OAc)_2_.^67^ Proton abstraction by O_2_^−^˙ facilitates the decarboxylation of radical cation 14A, resulting in radical 14B. When quinoxalin-2(1*H*)-one is added to by radical 14B, the nitrogen radical intermediate 14C is produced. After a second radical 14B attacks 14C, intermediate 14D is created.^68^ The cyclized product is then delivered *via* intramolecular nucleophilic substitution and aniline elimination^69^ (Scheme 14).

Selected instances of bond-forming reactions, including C–C, C–N, and C–O couplings under visible light irradiation, that are accomplished using synergistic photoredox-nickel catalysis are shown in Table 1. This dual-catalytic approach can be applied to a wide range of substrates and functional groups. Table 2 shows the critical analysis of representative reaction classes in Ni/photoredox dual catalysis, highlighting photocatalysts, nickel species, advantages, and key challenges.

**Table 1:** Some chemical reactions by combined photoredox and nickel catalysis

Entry	Photocatalysts	Light source	Reactants/starting materials	Products	References
1	4CzIPN	Visible light	Dihydropyridines, (hetero)aryl bromides	(Hetero)aryl amides	39
2	4CzIPN	Blue LED	Alkyl halides, vinyl bromides	Allyl carbamates	52
3	Ru-based photocatalyst	High-energy visible light	Iodoacetanilide, alkenes	Indoline	63
4	4CzIPN	High-energy visible light	Alkyl aziridines, (hetero)aryl iodides	β-Phenethylamine	70
5	4CzIPN	Visible light	α-Amino-oxy acids, alkene	1,2-Aminoarylation	34
6	4CzIPN	Blue LED	Deoxyribosyl acids, aryl/heteroaryl bromides	Aryl/hetero aryl-*C*-nucleosides	35
7	Organometallic iridium(iii) complex	Blue LED	Terminal alkyne, tertiary alkyl oxalates, aryl bromide	*Syn*-selective trisubstituted alkene	71
8	Ir^III^ Photocatalyst	Visible light	Terminal alkynes, alkyl carboxylic acids, aryl bromides		72
9	PCET	Visible light	Aryl bromides, amines	Aryl amines	73
10	Ir^III^ Photocatalyst	Visible light	Aldehydes, 1,3-dienes	Homoallyic alcohols	61
11	4CzIPN		Olefins, amides		74
12	Ir^III^ Photocatalyst	Visible light	Oxetane buiding block	Aryl aminooxetanes	36
13	Ir^III^ Photocatalyst	Visible light	(Hetero)aryl chlorides, ethers	C(sp^3^)–H arylation products, benzaldeyde	75
14	Ru-based photocatalyst	Blue LED light	*N*-aryl glycines, quinoxalin-2(1*H*)-ones	Tetrahydroimidazo[1,5-*a*] quinoxalin-4(5*H*)-ones	66

**Table 2:** Critical analysis of representative reaction classes in Ni/photoredox dual catalysis, highlighting photocatalysts, nickel species, advantages, and key challenges

Reaction class	Photocatalyst	Nickel catalyst	Key advantage	Limitations/challenges
C–N coupling (Scheme 1, carbamoyl radical to aryl bromide)	Organic photocatalyst (SET)	Ni^0^/Ni^II^ cycle	Mild conditions, broad amide precursors	Substrate constraints: requires careful matching of radical precursor & aryl halide
C–C bond formation (Scheme 2)	Ir-photoredox catalyst	Ni^0^/Ni^I^/Ni^II^ intermediates	Access to *β*-phenethylamines, stereocontrol	Multiple possible radical pathways; labeling errors corrected (2H/2I) highlight mechanistic complexity
C–O coupling (Scheme 4)	Ir(ppy)_3_	Ni^II^ intermediate	Efficient for aryl–O bond formation	Requires expensive Ir photocatalyst; organic alternatives suggested
Hydroalkylation & aryl–alkylation (Schemes 7–9)	Organic dyes	Ni^0^/Ni^I^/Ni^III^	Visible light, sustainable, avoids noble metals	Competing side reactions (over-reduction, radical recombination)
Enantioselective transformations (Scheme 10)	Dual PC/Ni system	Chiral Ni complexes	High enantioselectivity potential	Catalyst stability, high cost, and limited substrate scope
Heterogeneous CdS–Ni dual system (Scheme 13)	CdS semiconductor	Ni complex anchored	Eliminates pre-functionalization, heterogeneous reuse	Scalability, metal leaching, and stability of CdS under long irradiation

## Conclusion

Ni-catalyzed photoredox reactions override the drawbacks of conventional techniques by providing a potent platform for the selective and effective production of C–C and C–heteroatom bonds. In line with green chemistry principles, this method improves reaction accessibility by using visible light as a renewable energy source. A better understanding of reaction mechanisms, the necessity for affordable photocatalysts, and substrate constraints are some of the obstacles that still need to be overcome. Subsequent investigations ought to concentrate on creating sophisticated nickel catalysts, investigating innovative photocatalytic systems, and utilizing computational techniques to maximize effectiveness. If these problems are resolved, Ni-photoredox catalysis may become a fundamental component of synthetic chemistry, which would be advantageous for the materials science, agrochemical, and pharmaceutical sectors.

## Future outlook

Nickel-photoredox catalysis is on track to evolve into a versatile and sustainable platform for modern synthesis. Future advances will center on robust catalyst design, improved stability, and greater use of cost-effective organic photocatalysts to reduce dependence on precious metals. Deeper mechanistic insights will enable predictive control, while expanding the scope to enantioselective transformations, late-stage functionalization, and C–H activation will enhance synthetic utility. Green protocols, solar-driven systems, and flow technologies will further improve scalability and environmental compatibility, positioning Ni/photoredox catalysis as a powerful tool for both academic and industrial applications.

## Author contributions

Faiza Manzoor: writing – original draft. Adnan Majeed: writing review & editing, software. Ahmad H. Ibrahim: resources. Muhammad Adnan Iqbal: conceptualization, resources, supervision. Asma Rehman: data curation. Sadia Aziz: formal analysis. Anam Shahzadi: software. Sabahat Fatima: visualization. Sana Ejaz: validation. Muhammad Shehroz Zafar: formal analysis.

## Conflicts of interest

The authors declare that they have no competing interests.

## Data Availability

No primary research results, software, or code have been included and no new data were generated or analyzed as part of this review.
